# CT and quantitative 19F MRI tracking of encapsulated mesenchymal stem cells in a peripheral arterial disease rabbit model

**DOI:** 10.1186/1532-429X-15-S1-P24

**Published:** 2013-01-30

**Authors:** Guan Wang, Yingli Fu, Steven Shea, Judy A Cook, Dara Kraitchman

**Affiliations:** 1 Radiology and Radiological Science, Johns Hopkins University, Baltimore, MD, USA; 2 Electrical and Computer Engineering, Johns Hopkins University, Baltimore, MD, USA; 3 Molecular and Comparative Pathobiology, Johns Hopkins University, Baltimore, MD, USA; 4 Center for Applied Medical Imaging, Siemens Corporation, Corporate Research and Technology, Baltimore, MD, USA

## Background

Nearly 12% of Americans suffer from peripheral arterial disease (PAD) and many are not eligible for conventional angioplasty or surgical bypass. Microencapsulated stem cell (SC) therapy offers a novel means to transplant allogeneic SCs to avoid immunorejection and enable image tracking. However, quantitative evaluation of capsule fate is elusive. Here we explore c-arm CT and 19F-MRI for serial evaluation of dual X-ray/MR-visible SC microcapsules (XMRCaps) in a rabbit PAD model.

## Methods

XMRCaps were produced using a modified alginate microencapsulation method impregnating perfluorooctylbromine (PFOB) and human or rabbit SCs. In vitro validation studies were performed in a phantom consisting of known quantities of XMRCaps. C-arm CT images (dynaCT, Siemens Artis Zee) were acquired and reconstructed at 0.46 mm^3^ voxel size. 19F MRI was acquired with a flexible, 4-channel Tx/Rx 19F coil (3D TrueFISP, Siemens Tim Trio, 4.1 ms TR; 2.0 ms TE; 32 averages) in the coronal plane. 1H MRI was acquired with the system body or body matrix coil.

For *in vivo* studies, c-arm CTs and MRIs were acquired at one, two, and three weeks after human SC (XenoSC) or rabbit SC (AlloSC) XMRCap intramuscular administration in the hindlimb of a PAD rabbit (n=4) using identical imaging parameters as the in vitro studies. To test the repeatability of 19F MRI, imaging sets were acquired twice on the same day in one rabbit with the 19F coil repositioned in between. To enhance repeatability, the fluorine coil was constrained to a fixed geometry. Reference PFOB markers were placed within the imaging field to enable quantitation of XMRCaps.

Segmentation of the injections sites in c-arm CT and 19F MRI was performed with a thresholding algorithm. Relative fluorine concentration was determined by averaging the integrated 19F signal intensity over the segmented volume after normalization to the 19F standards.

## Results

**Figure 1:**
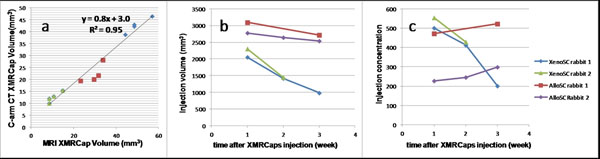
a) Correlation between MRI and CT volume of the 200 (blue), 100 (red) and 50 (green) XMRCaps. b) The XMRCaps injection volume in 19F MRI images at one, two and three weeks after delivery. The AlloSC rabbits have rabbit SCs and XenoSC rabbits has human SCs. c) Relative normalized fluorine concentration corresponding to the segmented volumes.

Calculated XMRCap volumes in CT and MRIs were highly concordant *in vitro* (Figure 1a). 19F MRI repeatability studies showed the volume and concentration errors of <3% and <6%, respectively. For the AlloSC rabbits, in vivo injection volume decreased 5±1% each week and concentration increased 12 ± 9% each week, while the XenoSC rabbits had volume and concentration decreases of 30 ± 2% and 35 ± 17%, respectively, each week (Figure 1b and c).

## Conclusions

MRI provides accurate assessment of XMRCap volumes with increased MRI volumes due to partial volume effects of the larger MRI voxel. In vivo volume changes of XMRCaps injection sites in the ischemic environment of the PAD rabbit could be quantified on MRI and CT. However, only MRI was able to demonstrate alterations in the fluorine concentration. In particular, decreases in XMRCap volumes with stable or increasing fluorine concentrations in animals that received allogeneic stem cells suggest better tolerance of allogenic microencapsuled cells than xenogenic stem cells.

## Funding

a grant from Siemens AG, NIH R33-HL089029, and the Maryland Stem Cell Research Foundation (2008-MDSCRFII-0399).

